# Long-term outcomes of cemented compared to uncemented femoral stems in total hip arthroplasty for displaced femoral neck fractures in elderly patients

**DOI:** 10.1007/s00068-024-02735-0

**Published:** 2025-01-24

**Authors:** Michael Axenhus, Ghazi Chammout, Paula Kelly-Pettersson, Sebastian Mukka, Martin Magnéli, Olof Sköldenberg

**Affiliations:** 1 Department of Clinical Sciences at Danderyd Hospital, Division of Orthopaedics, Karolinska Institutet, Stockholm, Sweden; 2 Department of Diagnostics and Intervention (Orthopaedics), Umeå University, Umeå, Sweden

**Keywords:** Elderly, Femoral, Cervical hip fractures, Hip replacement, Hip arthroplasty, RCT

## Abstract

**Background:**

Total hip replacement (THR) is commonly used for active and lucid elderly patients with displaced femoral neck fractures (FNF). Historically, cemented stems have been favoured, demonstrating superior early outcomes. Controversy still exists regarding the use of cemented or uncemented stems in the most active group of patients with FNF and there is a need for extended follow-up studies to assess long-term outcome of cemented and uncemented stem results.

**Methods:**

A 4 and 10-year follow-up was conducted on a single-centre, single-blinded, randomized controlled trial. Patients aged 65–79 years with an acute displaced FNF (Garden III–IV) were included, and surgeries were performed between 2009 and 2014. The study was terminated after an interim analysis indicated that the total number of early hip-related complications was substantially higher in the uncemented group. Baseline and follow-up assessments included hip-related complications, reoperations, health-related quality of life scores, Harris hip score and pain ratings.

**Results:**

In total, 69 patients were randomized. At 4 years, there were 8 complications in the uncemented group and 2 complications in the cemented groups. The uncemented group had several periprosthetic fractures and dislocations necessitating revisions in several cases. From 4 to 10 years, the cemented group showed a single periprosthetic fracture, while none occurred in the uncemented group. The total number of complications during the study period were 8 in the uncemented group and 3 in the cemented group. The median Harris hip score for the uncemented group remained consistent at 81 for both the 4- and 10-year follow-ups. In contrast, the cemented group showed scores of 92 and 93 at the respective 4- and 10-year follow-ups, with no statistically significant difference between the two groups. Health-related quality of life and pain ratings were similar between groups throughout the study.

**Conclusion:**

Our study presents a 10-year follow-up of uncemented femoral stems in THR for elderly FNF patients. Our findings not only underscore the importance of cautious decision-making in selecting patients for uncemented implants, but also highlight that most patients suitable for THR would benefit from a cemented arthroplasty to avoid an increased risk of short-term complications.

## Introduction

Total hip replacement (THR) is a treatment option for lucid older patients who have sustained a displaced femoral neck fracture (FNF) [1]. Historically, the choice between cemented and uncemented stems in hip arthroplasty for FNF patients has leaned towards cemented fixation. Cemented stems have demonstrated superior outcomes in pain relief, walking ability, reduced dependence on walking aids, and improved activities of daily living [2]. Moreover, they have exhibited a lower incidence of complications, including periprosthetic fractures, compared to their uncemented counterparts [3]. However, uncemented fixation remains widespread due to the risk of bone cement implantation syndrome, a concern associated with cemented stems. Bone cement implantation syndrome, although rare, does not occur in uncemented stems making this approach preferable for some clinicians despite the associated higher incidence of periprosthetic fractures [4].

Reports on modern uncemented, hydroxyapatite-coated femoral stems have presented encouraging early results regarding the use and risks of uncemented femoral stems [5–7].

In the present study, we hypothesized that an uncemented, proximally porous, and hydroxyapatite-coated femoral stem used in THR for displaced FNF would not be associated with more adverse perioperative and postoperative events compared to a THR using a cemented stem [8]. Additionally, we hypothesized that the health-related quality of life for patients would be equivalent at 2 years. This study is a follow up study of the cemented compared to uncemented femoral stems in total hip replacement for displaced femoral neck fractures in the elderly (CHANCE) trial. The initial power analysis for the study recommended a total of 140 included patients. However, a planned mid-term review found that the total number of early hip-related complications was substantially higher in the group who had undergone THR with uncemented femoral stems, resulting the termination of the study after inclusion of 69 patients [9].

In this follow-up study we describe the outcomes over a 10-year period with the same cohort. We aim to provide invaluable insights into the long-term outcome of a randomized controlled trial comparing cemented and uncemented stems for THR in active and lucid elderly patients with displaced FNF.

## Methods

### Trial design, settings, and location

Uncemented **C**ompared to cemented femoral stems in **H**ip **A**rthroplasty for displaced femoral **N**eck fractures in the elderly: A randomized **C**ontroll**E**d trial (CHANCE) trial was a single-centre, single-blinded, randomized controlled trial conducted between 2009 and 2023 (inclusion period: September 2009 to March 2014) at the Orthopaedic Department of Danderyd Hospital, Stockholm, Sweden. The CHANCE trial adhered to the principles of good clinical practice (ICH-GCP) and the CONSORT statement. The trial protocol has been published [8].

### Study subjects and eligibility criteria

Patients with a displaced femoral neck fracture (Garden III–IV) admitted to Danderyd Hospital during the inclusion period were screened for participation. Informed oral and written consent was obtained from willing participants. Inclusion criteria comprised surgery within 48 h, age between 65 and 79 years, intact cognitive function, and independent ambulation with or without walking aids. Patients with pathological fractures, rheumatoid arthritis, symptomatic osteoarthritis, or deemed unsuitable for THR due to severe comorbidities were excluded.

### Data collection and follow-up

Baseline assessments included interviews regarding living conditions, mobility, activities of daily living (ADL) status, and health-related quality of life measured by EQ-5D. Follow-up evaluations occurred at 3, 12, 24 months, and at 4 and 10 years after the surgery. Patients unable to attend in person were interviewed by phone or sent questionnaires for completion. Research nurses were responsible for the gathering of data throughout the study period. Hip-related complications were identified through the unique Swedish personal identity number, cross-referencing digital medical charts, the Swedish Hip Arthroplasty Register, and the Swedish Patient Registry.

### Surgery

Experienced surgeons performed the operations, using a direct lateral approach with the patient in the lateral decubitus position [8]. The cemented group received the modular CPT (Zimmer, Warsaw, Indiana, USA) collarless, polished, tapered femoral component made from cobalt-chromium. The uncemented group received the Bi-Metric stem (Biomet, Warsaw, Indiana, USA), a tapered femoral stem with a proximally coated, plasma-sprayed, commercially pure, titanium surface, was employed. A 32 mm cobalt-chromium head was used for all patients. For the acetabular component, a cemented XLPE cup and vacuum-mixed low-viscosity cement with gentamicin were used. Low-molecular-weight heparin was administered postoperatively for at least 10 days, and antibiotic prophylaxis was followed.

### Endpoints

Primary endpoints, evaluated at 4- and 10-years post-surgery, included the prevalence of all hip-related complications and reoperations and changes in health-related quality of life assessed with EQ-5D index. Secondary endpoints encompassed overall mortality, hip function assessed using Harris hip score (HHS) and pain in the operated hip measured with the numerical pain rating scale (NPRS).

### Randomization and blinding

In the CHANCE trial, patients were block-randomized in groups of 10 with a 1:1 ratio, ensuring equal distribution between cemented and uncemented stem groups. Sealed envelopes were used for randomization which was stratified by sex to maintain a balanced gender representation in both groups. The patients remained blinded to their treatment assignment up until the 2-year follow-up. To confirm the integrity of blinding, patients were informed about their assigned treatment at the 2-year follow-up. The surgeons and staff were not blinded for the duration of the study.

### Statistics

The CHANCE trial adhered to the intention-to-treat principle, whereby patients remained in their initially randomized groups regardless of any subsequent surgical interventions. Descriptive statistics, including means, medians, and standard deviations, were used to characterize patient attributes and outcome variables. Fisher’s exact test was utilized to assess the first primary endpoint, while Student’s t-test and Levene’s test were applied for comparisons involving the second primary endpoint and other functional outcomes. Confidence intervals of 95% were calculated and p-values ≤ 0.05 were considered statistically significant. SPSS version 22 was used for all analyses.

### Ethics and registration

The study adhered to the ethical guidelines outlined in the Helsinki Declaration and received approval from the Ethics Committee at the Karolinska Institute (2009/97 − 31/2). The study has been registered on Clinicaltrials.gov (identifier: NCT02247791).

## Results

### Patient flow and baseline data

**Fig. 1:**
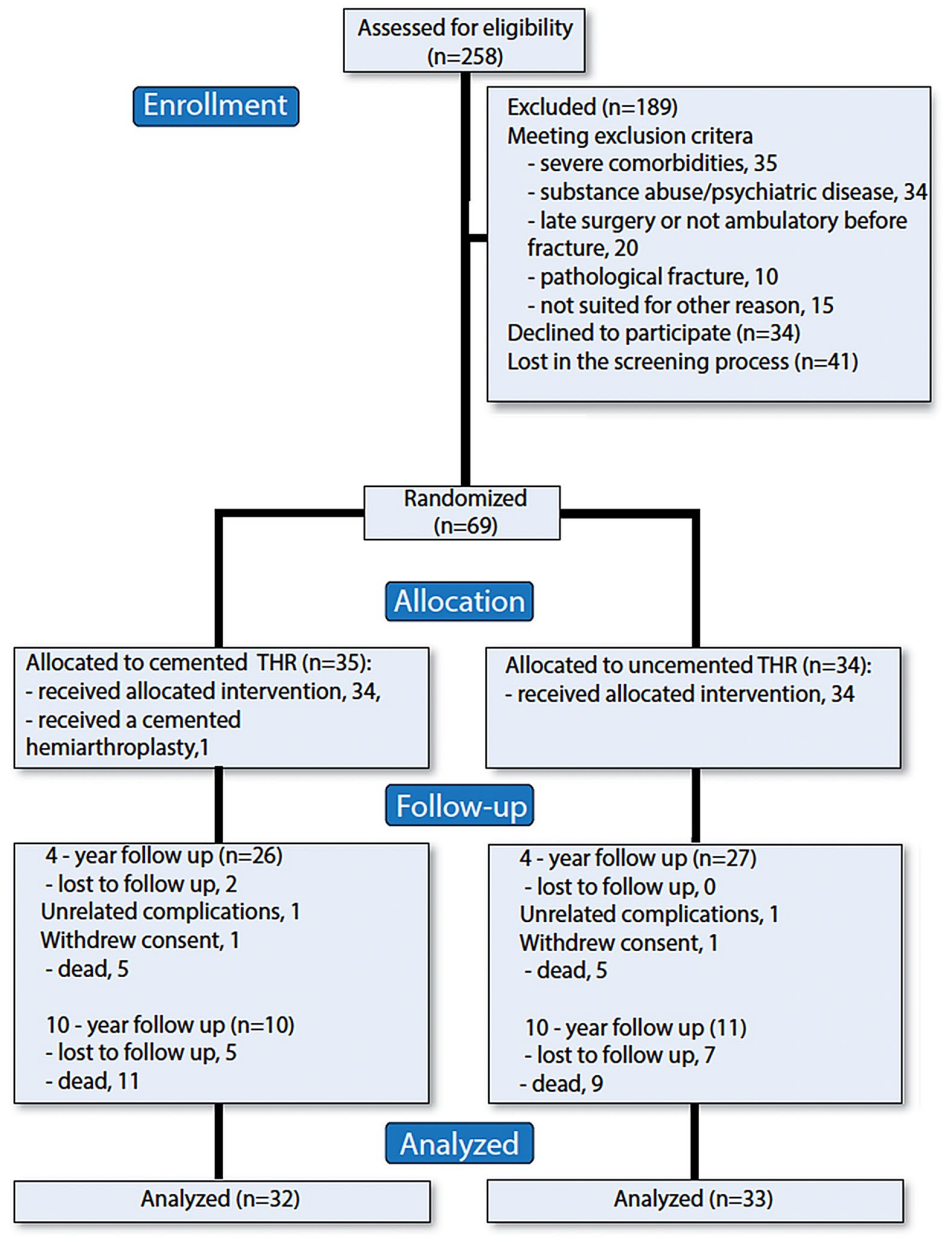
CONSORT flow diagram

Between September 2009 and March 2014, 258 patients with displaced FNF were admitted to the Orthopaedic Department at Danderyd Hospital. Of these, 69 patients met the inclusion criteria and were recruited to the study. The mean age was 73 years, with 47 female patients. 53 and 21 patients remained at the 4- and 10-year follow up, respectively (Fig. 1).

### Primary endpoints

The health-related quality of life, measured using EQ-5D, remained similar between the two groups throughout the study period (Table 1).

**Table 1:**
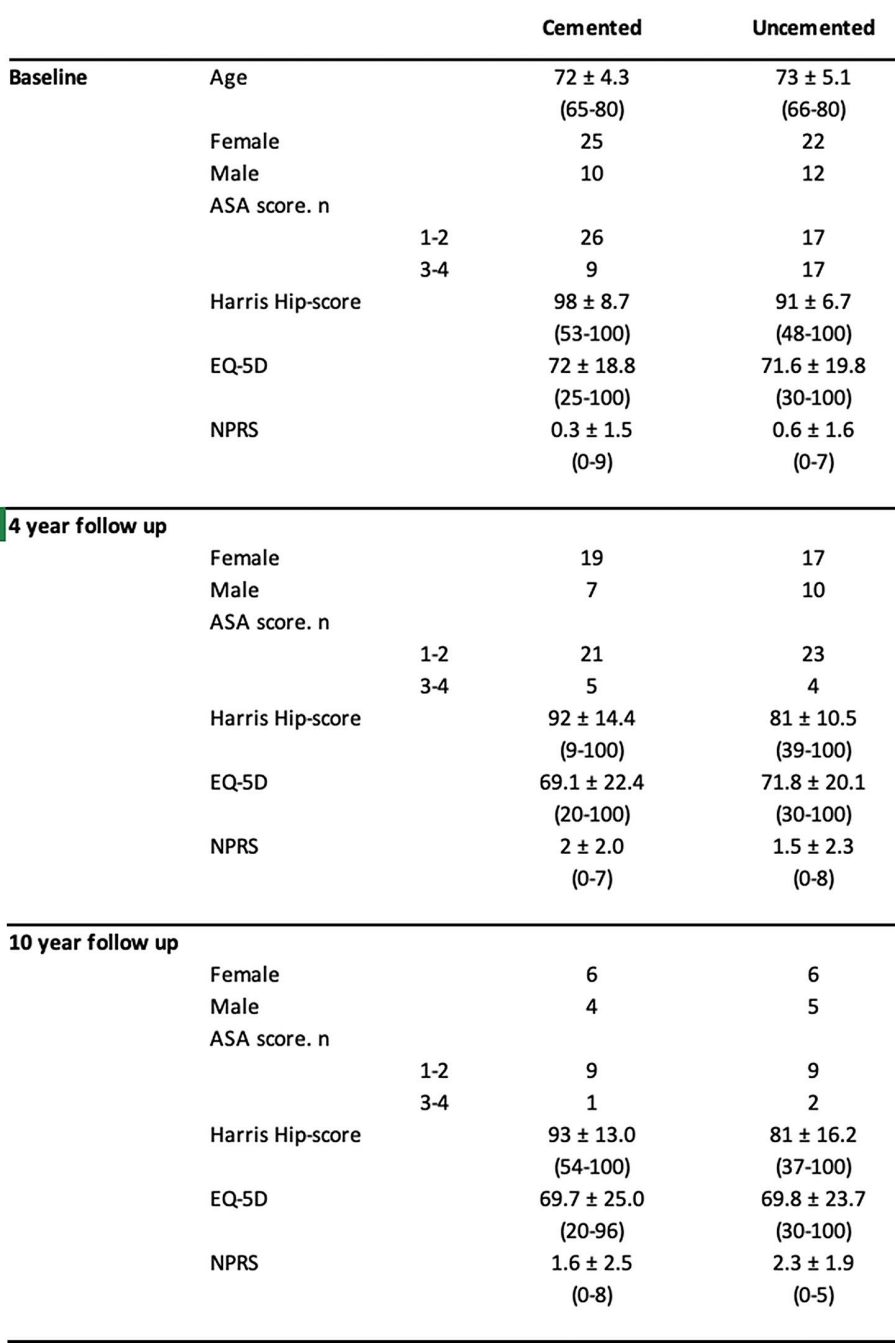
Age at baseline, female to male distribution, ASA score, median Harris hip score, mean EQ-5D and mean numerical pain rating scale (NPRS) at the 4- and 10-years follow up

At 4-years post-surgery, complications had occurred in 9 patients, 1 case in the cemented group and 8 in the uncemented group (relative risk = 7, 95% CI: 1–40; *p* = 0.006).

Among those with uncemented stems, four periprosthetic fractures occurred. Two periprosthetic fractures were treated with ORIF, one with a stem revision and one was treated non-surgically. One patient treated with ORIF later experienced a dislocation, necessitating a cup revision. Two other patients also experienced dislocations, of which one required a stem revision. One patient experienced a femur fracture on the operated side that was treated with ORIF. In the cemented group, there were one case of dislocation and one case of periprosthetic fracture which required plate fixation. At 10 years after surgery, one patient in the cemented group experienced a periprosthetic fracture, which was treated non-operatively. In contrast, no complications were observed in the uncemented group during this extended follow-up period (Table 2).

**Table 2:** Complications at 4- and 10- years follow up

	Up to 4 years		4 to 10 years	
	Cemented	Uncemented	Cemented	Uncemented
Dislocation	1	3	0	0
Femur fracture(operated side)	1	1	0	0
Periprosthetic fracture	0	4	1	0
Total number complications	2	8	0	0
Nonoperative treatment	0	1	1	0
Stem revision	0	2	0	0
ORIF	1	3	0	0
Cup revision	0	1	0	0
Closed reduction	1	1	0	0

**Fig. 2:**
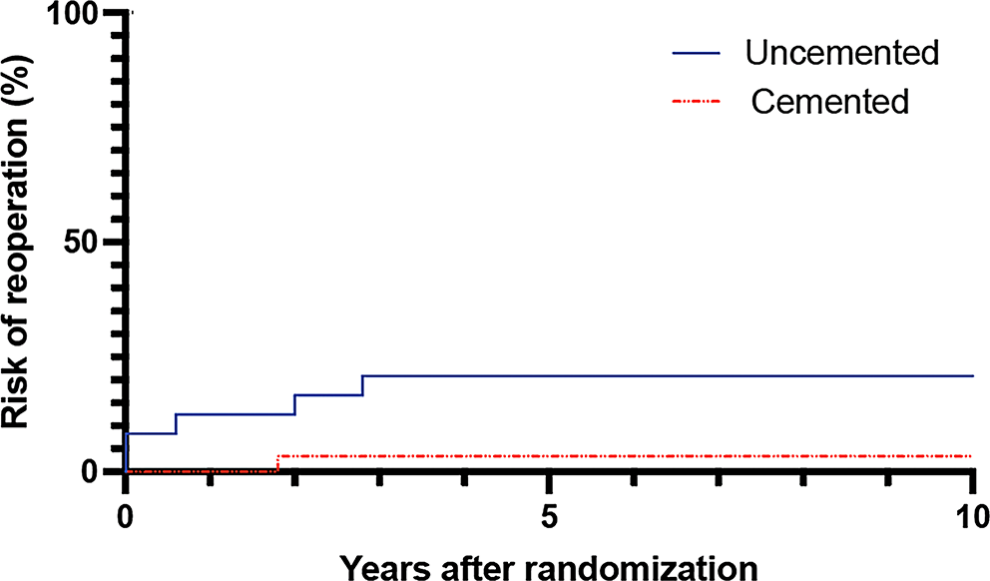
Kaplan-Meier graph showing a 21% risk of reoperation for uncemented femoral stems compared to a 5% risk of reoperations for cemented stems during a 10 year follow up period

There was a 21% risk of reoperation for uncemented femoral stems compared to a 5% risk of reoperation for cemented stems (*p* < 0.001) during the 10 years follow up (Fig. 2).

### Secondary endpoints

**Fig. 3:**
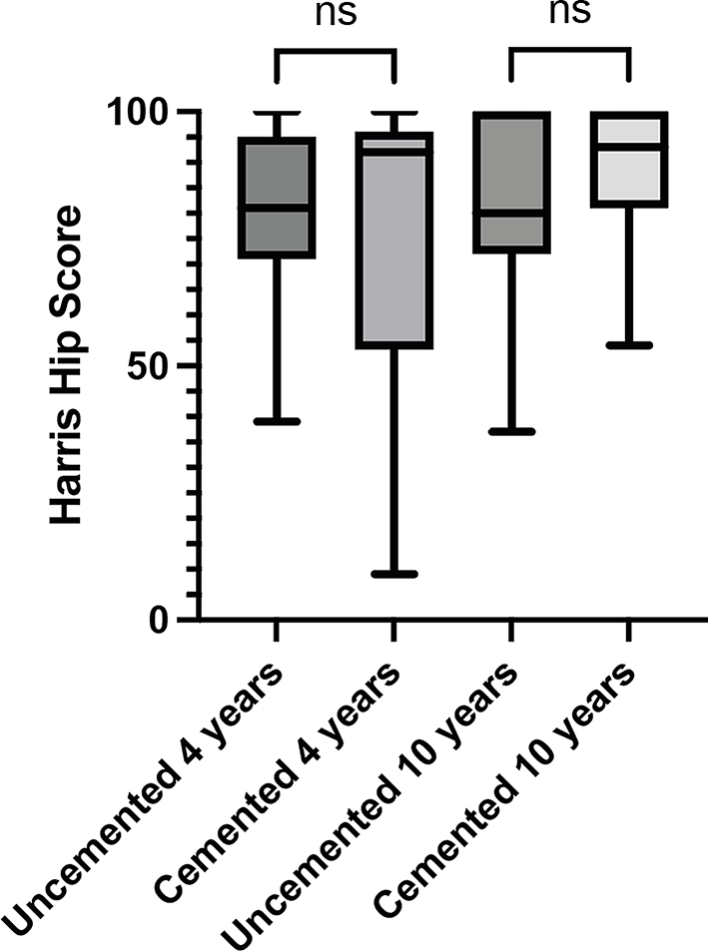
Median Harris hip score at 4- and 10-year follow up

The median HHS score was higher in the cemented group after both 4 and 10 years compared to the uncemented group (Fig. 3). NPRS was lower in the uncemented group at the 4-year mark but rose and was higher at the 10-year point in the uncemented group compared to the cemented group. None of these variances reached statistical significance.

At the 4 years follow up point, 10 patients had died. 5 in the cemented group and 5 in the uncemented group. The registered causes of death were cancer, cardiac arrest, infections and one intoxication. At the 10-year follow-up, a total of 30 patients had died. 16 in the cemented and 14 in the uncemented group. There was no statistical difference in mortality at the 4-year and 10-year follow up (Table 3).

**Table 3:**
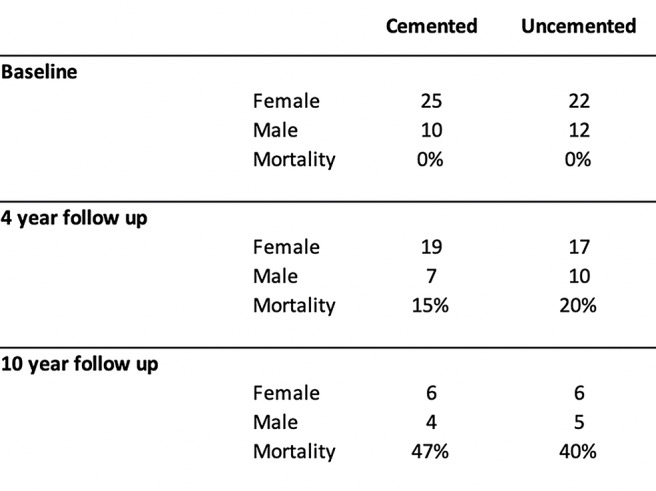
Surviving patients and associated mortality at 4- and 10- year follow up

## Discussion

This study presents a 10-year follow-up of an RCT comparing cemented and uncemented femoral stems in THR for elderly FNF patients. Short-term complications with uncemented stems are evident, but long-term performance showed promise. This nuanced understanding aids clinicians in informed decision-making and provides evidence for meta-analyses.

The strengths of the CHANCE trail study included its randomized controlled design, allowing for a robust comparison between the two techniques. Unlike previous studies that have primarily focused on hemiarthroplasty, our trial evaluated modern cemented and uncemented hydroxyapatite-coated stems in the context of THR. The trial utilized intention-to-treat analysis and stratified randomization by sex to ensure a balanced representation, enhancing the reliability of our findings. However, it is crucial to acknowledge the limitations of our study. One major constraint was the premature termination due to a high rate of complications in the uncemented group, leading to a smaller sample size than initially intended. Furthermore, there was no stratification of patients based on ASA class or femur Dorr type. Despite these limitations, our findings provide insights into the complexities of THR for displaced FNF in elderly patients.

Despite obvious limitations in long-term follow-up due to early termination, our study still provide insight in the evolving landscape of hip arthroplasty treatments. Unlike previous trials which have focused on hemiarthroplasty [7, 10–13], the CHANCE trial is one of few blinded randomized clinical trials designed with a THR approach using modern hydroxyapatite-coated stems. Our results were obtained despite using a polished tapered cemented stem linked to an increased risk for periprosthetic femoral fractures [14–17].

Previous studies have primarily favoured cemented fixation, emphasizing better mobility, reduced periprosthetic fractures, and less thigh pain [2]. However, the cementing process carries risk and can cause cardiac arrythmia and cardiovascular collapse [18], making uncemented fixation an appealing treatment option in elderly patients with comorbidities. However, a recent systematic review and meta-analysis has questioned the impact of the type of stem fixation [19]. The introduction of modern hydroxyapatite-coated implants revived the interest in uncemented fixation, promising shorter surgery duration and potentially reduced risks of infection and with the aim of the risk of reducing aseptic loosening as a potential long-term complication [20]. A pilot study conducted at our department found that there was no increase in the complication rate in elderly patients with osteoporotic fractures treated with uncemented modern stems [6].

Our findings at the 4-year mark demonstrated a significantly higher rate of hip-related complications in the uncemented group, validating the concerns raised by our 2-year results. Notably, these complications were diverse, ranging from periprosthetic fractures to dislocations, necessitating various interventions including stem revisions, plate fixations, and cup revisions. In contrast, the cemented group exhibited a more stable outcome, only two reported complications during the 4-year follow-up period.

Interestingly, our 10-year follow-up data presented a different narrative. The uncemented group, which experienced several complications at 4 years follow-up showed no additional issues. In contrast, the cemented group encountered a single periprosthetic fracture, managed non-surgically. Nevertheless, our results reinforce the importance of long-term evaluation in orthopaedic research.

Recent literature suggests that the long-term benefit of either fixation method in THR for FNF is uncertain. A study of 17,491 patients found that cemented femoral fixation had a higher incidence of medical complications, although this was due to significant differences in comorbidities between groups [21]. A smaller study which used a specific system has also demonstrated no difference in long-term outcomes [22]. A meta-analysis on cemented versus uncemented stems for THR found that there was no difference in mid-term outcome for either group [23]. Our findings underscore the previously described challenges associated with introducing press-fit stems into osteoporotic bone [24, 25]. Furthermore, the potential benefit of antibiotic-loaded bone cement in preventing periprosthetic joint infection is an aspect to consider in treating patients with increased body mass index and an elevated number and severity of comorbidities [26, 27].

Our study’s results have implications for the broader orthopaedic community. Firstly, this extended follow-up highlights the need for careful consideration when selecting implant types for elderly FNF patients. Cemented stems demonstrated superior stability in the short term and similar long-term results as uncemented stems, this nuanced understanding is important for clinicians making informed decisions, especially for patients with extended life expectancies. Furthermore, our data enriches the pool of evidence available for meta-analyses and systematic reviews. As the orthopaedic field advances, incorporating these extended outcomes into meta-analyses enhances the robustness and accuracy of conclusions drawn from pooled data.

In conclusion, our 10-year follow-up study contributes valuable insights into the long-term outcomes of cemented and uncemented femoral stems in THR for elderly FNF patients. While both techniques have their merits, our data suggests fewer complications with cemented stems in the medium term but also suggests stable performance of uncemented stems in the long term. Our findings underline the importance of cautious decision-making in selecting patients for uncemented implants and emphasizes that most patients suitable for THR would benefit from a cemented arthroplasty to avoid the increased risk for short-term complications.

## Data Availability

No datasets were generated or analysed during the current study.

## Abbreviations

ADL: activities of daily living
FNF: femoral neck fractures
HHS: Harris hip score
NPRS: numerical pain rating scale
THR: total hip replacement
